# Editorial: Mechanisms of orofacial pain

**DOI:** 10.3389/fpain.2024.1496188

**Published:** 2024-11-15

**Authors:** Shivani B. Ruparel, Armen N. Akopian

**Affiliations:** ^1^ Center for Pain Therapeutics and Addiction Research, School of Dentistry, University of Texas Health San Antonio, San Antonio, TX, United States; ^2^ Department of Endodontics, School of Dentistry, University of Texas Health San Antonio, San Antonio, TX, United States; ^3^ Department of Pharmacology and Physiology, School of Medicine, University of Texas Health Science Center at San Antonio, San Antonio, TX, United States

**Keywords:** orofacial pain, peripheral mechanisms, trigeminal neurons, non-neuronal cells, immune cells

**Editorial on the Research Topic**
Mechanisms of orofacial pain

Orofacial pain affects nearly 27% of the population (1, 2). While acute orofacial pain serves a protective function, chronic orofacial pain—lasting more than 3 months—lacks a clear biological purpose (3). Chronic orofacial pain encompasses a range of challenging conditions, including trigeminal neuralgia, temporomandibular joint and muscle disorders (TMD), odontogenic issues, and oral afflictions such as oral cancer, mucositis, and burning mouth syndrome. These conditions can have significant long-term impacts on key head and neck functions, including aesthetics, speech, eating, sleeping, and overall psychosocial well-being. Diagnosing and managing chronic orofacial pain presents numerous challenges due to its complex pathophysiology and the limited treatment options beyond opioids and invasive procedures.

For decades, research on the basic mechanisms of orofacial pain progressed slowly, with studies largely being descriptive and lacking in-depth experimental analysis. However, recent years have seen a surge in research activity, with over 1,000 papers published annually on orofacial pain. Significant advancements have been made in our understanding of the mechanisms underlying orofacial pain, driven by the application of novel technologies for identifying cellular and genetic plasticity, the development of clinically relevant animal models, and the in-depth analysis of clinical samples. Importantly, these advances have been fueled by innovative and groundbreaking ideas.

This Special Issue of “*Mechanisms of orofacial pain*” in *Frontiers in Pain Research* features six articles that highlight recent advances in our understanding of the peripheral mechanisms of orofacial pain, focusing on areas such as mechanisms, diagnosis, and interventional modalities. While it is impossible to cover all aspects of orofacial pain in a single set of reviews, we have endeavored to present a broad scope of research, with an emphasis on the application of novel technologies and research ideas to deepen our understanding of this condition. Written by world experts, these reviews are timely, accessible to a broad audience, and promise to be engaging reads. The reviews follow a “molecule to disease” presentation style and employ a “bench to bedside” methodological approach.

There is a consensus that orofacial pain originates from tissue and extracellular matrix damage in the periphery, which alters the properties of sensory afferent nerves and the characteristics of sensory neuronal and non-neuronal cells in the trigeminal ganglia (TG). This plasticity manifests as sensitization of sensory neurons, leading to increased excitability at peripheral nerve terminals and enhanced neurotransmission at central terminals. These findings underscore the importance of understanding changes in cellular and genetic plasticity in affected tissues. Omic technologies have provided unprecedented insights into both cellular and genetic plasticity under both normal and pathological conditions. For instance, Dr. Ruparel's group was the first to report sex-dependent changes in the transcriptomic profile of lingual sensory neurons during a pathological orofacial condition, specifically tongue cancer (4). Building on this, we proposed novel high-resolution approaches for using single-cell RNA sequencing to identify non-neuronal cell types in the TG of naïve mice and their gene expression profiles (Mecklenburg et al.). The increased sensitivity of these approaches enabled the identification of several types of macrophages and neutrophils in the TG, and revealed that resident macrophages within the ganglia possess unique gene profiles, distinguishing them from other known macrophage types (Mecklenburg et al.). Additionally, this study indicated that neutrophils reside in the dura surrounding the TG (Mecklenburg et al.).

While our study determined the genomic profiles of TG cell types, understanding the functions of these cells and the genes they express in the ganglia is crucial. Wang et al. reviewed function of vanilloid subtype 1 (TRPV1), transient receptor potential ankyrin subtype 1 (TRPA1), acid-sensing ion channel 3 (ASIC3), and the P2X3 receptor in mediation of orthodontic pain. Ronan et al. reviewed approaches for defining the functions of whole neurons innervating tissues in the head and neck area. Specifically, they discussed how a combination of transcriptomic analysis, anatomical studies, and cell-type-specific *in vivo* imaging can precisely identify the biological roles of sensory neurons innervating different structures of the tooth, such as the pulp, dentin, enamel, and root area (Ronan et al.). Perry et al. review expands on this topic, discussing the innervation of other head and neck tissues, including the masticatory muscles and TMJ, which are central to debilitating orofacial conditions like TMJD and myogenous TMD. They also highlight significant knowledge gaps related to the identities and functions of TG sensory neurons innervating other head and neck tissues, particularly joints and muscles (Perry et al.).

The precise identification of cellular and genetic plasticity in both neuronal and non-neuronal cells will ultimately lead to the construction of a comprehensive interactome network for various orofacial pain conditions (5). This will not only facilitate the discovery of disease-associated therapeutic targets but also emphasize the importance of targeting peripheral non-neuronal cells as whole in managing chronic orofacial pain. In this regard, Ren et al. reviewed the outcomes of mesenchymal stem cell treatments for pain, comparing the properties of stem cells derived from different tissues. Their review also discussed the role of immune cells and the endogenous opioid system in the analgesic and anti-nociceptive properties of orofacial mesenchymal stem cells (Ren et al.). Mesenchymal stem cell therapy holds great promise for managing chronic orofacial pain as well as bone remodeling (Wang et al.), given the significant advancements in methods and procedures to treat patients experiencing orofacial pain (Ren et al.).

Orofacial pain is a complex and multifaceted phenomenon influenced by both biological and psychosocial factors (6). To effectively study the basic mechanisms of various pain conditions—whether acute, inflammatory, neuropathic, cancer-related, arthritic (joint), muscle, or postoperative—it is crucial to employ translationally relevant animal models that can enhance the reliability of mechanistic findings. The development of clinically relevant orofacial pain models has been challenging due to the heterogeneity of many clinical pain conditions in the head and neck area, and the lack of precise pathophysiological data, particularly at the molecular and cellular levels. Barjandi et al. reviewed the clinical and mechanistic aspects of masticatory myalgia (TMD-M), the most common cause of non-odontogenic orofacial pain. Their review discusses the epidemiological, diagnostic, and etiological aspects of TMD-M, as well as the potential risk factors for its transition from acute to chronic (Barjandi et al.). This review not only enhances clinical knowledge about TMD-M but also helps bridge the gap in generating relevant animal models to study this debilitating and heterogeneous pain condition (Barjandi et al., 7).

In conclusion, we hope that this collection of reviews, written by leaders in the field, provides a timely and insightful read. This set of reviews also aim to promote interdisciplinary collaborations, inspire new research ideas, and contribute to a deeper understanding of orofacial pain mechanisms, ultimately leading to the development of new therapeutic strategies and approaches in prevention, diagnosis, and management to improve prognosis and reduce patient suffering from orofacial pain.
